# Clinical characteristics and predictive model of pulmonary tuberculosis patients with pulmonary fungal coinfection

**DOI:** 10.1186/s12890-023-02344-4

**Published:** 2023-02-07

**Authors:** Hongxuan Yan, Li Guo, Yu Pang, Fangchao Liu, Tianhui Liu, Mengqiu Gao

**Affiliations:** 1 Beijing Chest Hospital, Capital Medical University/Beijing Tuberculosis and Thoracic Tumor Research Institute, Postal No 9, Beiguan Street, Tongzhou District, Beijing, 101149 People’s Republic of China; 2 Xuzhou Medical University, Jiangsu, China

**Keywords:** Pulmonary fungal infection, Pulmonary tuberculosis, Coinfection, Predictive model

## Abstract

**Background:**

In clinical settings, pulmonary tuberculosis (PTB) patients were often found to have pulmonary fungal coinfection. This study aimed to assess the clinical characteristics of patients suffering from coinfection with TB and pulmonary fungal and construct a predictive model for evaluating the probability of pulmonary fungal coinfection in patients with pulmonary tuberculosis.

**Methods:**

The present case–control study retrospectively collected information from 286 patients affected by PTB who received treatment from December 6,2016- December 6,2021 at Beijing Chest Hospital, Capital Medical University. As control subjects, patients with sex and address corresponding to those of the case subjects were included in the study in a ratio of 1:1. These 286 patients were randomly divided into the training and internal validation sets in a ratio of 3:1. Chi-square test and logistic regression analysis were performed for the training set, and a predictive model was developed using the selected predictors. Bootstrapping was performed for internal validation.

**Results:**

Seven variables [illness course, pulmonary cavitation, broad-spectrum antibiotics use for at least 1 week, chemotherapy or immunosuppressants, surgery, bacterial pneumonia, and hypoproteinemia] were validated and used to develop a predictive model which showed good discrimination capability for both training set [area under the curve (AUC) = 0.860, 95% confidence interval (CI) = 0.811–0.909] and internal validation set (AUC = 0.884, 95% CI = 0.799–0.970). The calibration curves also showed that the probabilities predicted using the predictive model had satisfactory consistency with the actual probability for both training and internal validation sets.

**Conclusions:**

We developed a predictive model that can predict the probability of pulmonary fungal coinfection in pulmonary tuberculosis patients. It showed potential clinical utility.

**Supplementary Information:**

The online version contains supplementary material available at 10.1186/s12890-023-02344-4.

## Introduction

Tuberculosis (TB) is an infectious disease caused by *Mycobacterium tuberculosis* (MTB), with pulmonary tuberculosis being the most common type, accounting for 80–90% of all cases [1–3]. Patients with Pulmonary tuberculosis infection are susceptible to combined infection due to other pathogens, including pulmonary fungal infection due to hypoimmunity, altered bronchial structure, and lung tissue injury [6]. Pulmonary fungal infection is a respiratory disease caused by various pathogens including yeast species like *Candida albicans* and molds like *Aspergillus fumigatus*. Studies worldwide have reported pulmonary fungal coinfections among PTB patients, with *Aspergillus* spp as the predominant pathogen [4–6]. Some others have reported *Candida albicans* as the most prevalent fungi spp [7, 10]. Regardless of aspergillosis caused by *Aspergillus,* candidiasis caused by *Candida albicans*, or other types of pulmonary fungal infections, similarities in clinical symptoms and radiological features with PTB are present, which can easily lead to misdiagnosis and contribute to a high rate of mortality [8, 31, 33, 35]. A study illustrated that up to 1 million people recovering from tuberculosis developed pulmonary fungal coinfections annually and were mostly misdiagnosed as cases of relapsed PTB [9]. However, at present, some clinicians do not pay enough attention to this problem [32]. Therefore, related studies on coinfection with PTB and pulmonary fungal infection are needed so that these patients may benefit from targeted antifungal agents promptly [36]. Some existing research on clinical features of coinfection with PTB and pulmonary fungal infection has been published but corresponding predictive models are scarce even though the nomogram is a reliable tool to create an intuitive graph based on a statistical predictive model to quantify the risk of a clinical event [11, 12]. Given this background, we aimed to build a predictive model for assessing the probability of pulmonary fungal coinfection in patients with PTB.

## Methods

### study design and patients

**Fig. 1:**
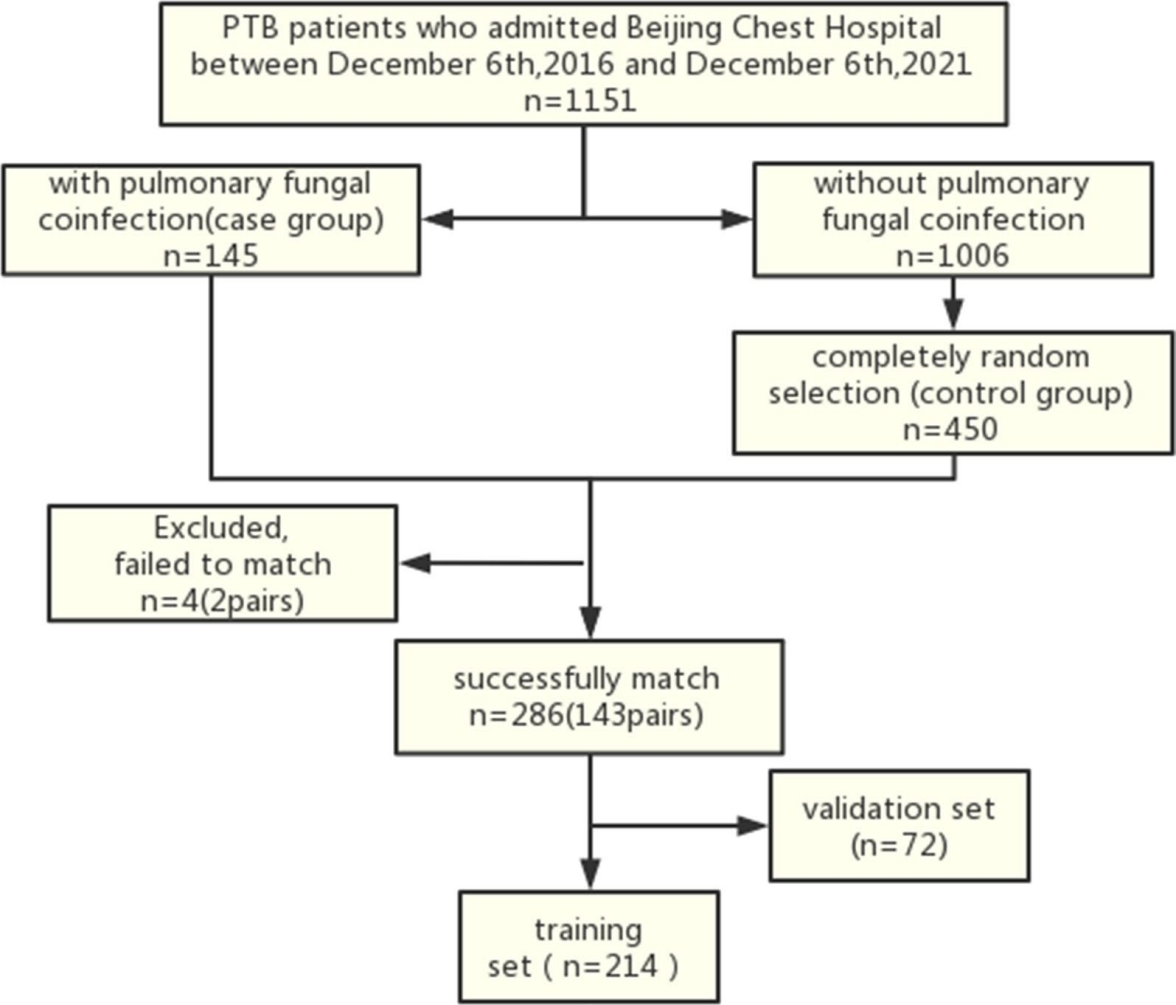
Study flow chart of patients selection

A retrospective case–control study was designed and conducted to identify the clinical characteristics of PTB patients with pulmonary fungal coinfection and develop a corresponding prediction model. First, we retrospectively retrieved data from 1151 PTB patients who had received treatment in Beijing Chest Hospital, Capital Medical University, from 2016.12.6–2021.12.6 (including 145 patients with pulmonary fungal coinfection who were assigned to the case group) using the electronic medical record system. Next, we randomly selected 450 PTB patients without pulmonary fungal coinfection as the control group. The two groups of patients (case:control) were matched by sex and address characteristics in a ratio of 1:1. Finally, 143 patients each (2 case subjects were excluded as they could not be matched) in the case group and control group were included. Subsequently, these 286 patients were randomly divided into a training set and an internal validation set in a ratio of 3:1. Univariate analysis and multivariate logistic regression analysis were performed in the training set to evaluate the probability of pulmonary fungal coinfection in PTB patients and construct a prediction model. A nomogram was developed using selected predictors. Bootstrapping was performed for internal validation. (Fig. 1).

### Inclusion and exclusion criteria

Case group: Eligible patients included those with confirmed PTB and pulmonary fungal infection who simultaneously meet the diagnostic criteria for tuberculosis according to the “Health Industry Standards of the People's Republic of China” issued in 2017 [13] and pulmonary fungal infection according to the “Expert Consensus on the Diagnosis and Treatment of Pulmonary Mycosis” proposed by the Infections Group of the Respiratory Society of the Chinese Medical Association in 2007 [14]. Other criteria were as follows: age, 16 years or older, and those who were hospitalized. Patients with missing information were excluded.

Control group: Eligible patients included those with confirmed PTB but without pulmonary fungal infection who only meet the diagnostic criteria for tuberculosis according to the “Health Industry Standards of the People's Republic of China” issued in 2017 [13] but not for pulmonary mycosis as stated in the “Expert Consensus on the Diagnosis and Treatment of Pulmonary mycosis” issued by the Infectious Diseases Group of the Respiratory Society of the Chinese Medical Association in 2007 [14]. Other criteria were as follows: age, 16 years or older, and those who were hospitalized. Patients with missing information and those with suspected pulmonary fungal infection were excluded.

Following are the diagnostic criteria for pulmonary fungal infection according to the “Expert Consensus on the Diagnosis and Treatment of Pulmonary Mycosis” proposed by the Infections Group of the Respiratory Society of the Chinese Medical Association in 2007: 1. at least present one host incidence risk factor. 2. Those who meet clinical characteristics of pulmonary mycosis {(1) Main clinical features: 1. invasive pulmonary aspergillosis with chest X-ray and Computed Tomography examination showing increased subpleural nodules or halo symptoms around the lesions in the early stages of the disease; chest X-ray and CT examination showing pulmonary cavity or a crescent sign 10–15 days after onset; 2. Pneumocyte pneumonia: chest CT examination showing hair glass lung interstitial infiltration. (2) Secondary clinical features: 1. continuous fever > 96 h with no significant improvement after aggressive antibiotic treatment; 2. symptoms and signs of a lung infection, including cough, expectoration, hemoptysis, chest pain, dyspnea, moist rales or pleural friction sound; 3. imaging examination showing new non-specific lung infiltration other than the main clinical features}, 3. Those presenting lung histopathological evidence. 4. Those with microbiological evidence {(1) direct microscopy of the endotracheal attractor or qualified sputum samples showing hyphae, and the same fungus is isolated two consecutive times in the culture. (2) BALF is detected by direct microscopy with a positive fungal culture. (3) Qualified sputum or BALF direct microscopy or culture showing *Cryptococcus neoformans*. (4) Positive for Crycoccus capsular polysaccharide antigen by latex coagulation. (5) The G-test is positive two consecutive times. (6) The GM-test is positive two consecutive times}.

### Candidate predictors

We reviewed the relevant literature on the clinical characteristics of coinfection with PTB and pulmonary fungal infection and based on the existing evidence [15–17, 27, 34, 37], the following patient data variables were collected as candidate predictors: 1. demographic features including age (> 65/ ≤ 65 years), sex (male/female), and address (north/south, considering that the pathogens and incidence rate of pulmonary fungal infection vary by area, which we classified as north and south rather than rural and urban); 2. personal histories such as smoking and drinking habits (yes/no, the “no” means “never”); 3. features of PTB including category (initial/recurrent), drug resistance (no/single drug resistance/other drug resistance, “single-drug resistance including MR or RR”, “other drug resistance including PDR or MDR or Pre-XDR or XDR”), and illness course (from the time of onset to the time of hospitalization); 4. imaging findings including pulmonary cavitation (yes/no) and pleural effusion (yes/no); 5. medication including regular anti-TB treatment (yes/no), use of broad-spectrum antibiotics for at least 1 week (yes/no), use of glucocorticoids for at least 2 weeks (yes/no), and chemotherapy or immunosuppressant use (yes/no); 6. therapeutic operations including invasive operation (yes/no, invasive operation refers to the operations requiring access to human sterile tissues, organs, vasculature or contact with skin mucosa for diagnosis and treatment, including arteriovenous catheterization, ventilator application, endoscopy, endotracheal intubation, tracheotomy, hemodialysis, puncture operations such as thoracentesis, bone marrow aspiration, lumbar puncture, abdominocentesis, and other interventional operations) and surgical history (yes/no); 7. comorbidities including bacterial pneumonia (yes/no), chronic bronchitis or chronic obstructive pulmonary disease or bronchiectasis or asthma (yes/no), silicosis (yes/no), interstitial lung disease (yes/no), hepatic injury (yes/no), renal inadequacy (yes/no), tumors or hematological diseases (yes/no), anemia (yes/no), and hypoproteinemia and diabetes (yes/no). All variables were collected at the time of hospital admission as baseline data except for the use of broad-spectrum antibiotics for at least 1 week and glucocorticoids for at least 2 weeks. These two excluded factors were collected in the period corresponding to 6 months prior to hospitalization to hospitalization.

### Statistical analysis

We used SPSS 25.0 and R 4.2.0 for statistical analyses which were all two-tailed, and a *P*-value < 0.05 represented statistical significance. All candidate predictors were categorical variables summarized as a frequency in counts and percentages. In the training set, the chi-square test or Fisher's exact test was used for conducting univariate analysis to compare differences in features between PTB patients with and without pulmonary fungal coinfection. Based on the results of the univariate analysis, significant variables with *P* < 0.05 were used as input in the multivariate logistic regression model, from which significant variables were deemed as predictive factors to establish the prediction model for assessing the probability of pulmonary fungal coinfection in PTB patients. Finally, a nomogram was constructed according to the model. The area under the curve (AUC) was calculated to assess the discrimination capacity of the model, and internal validation was performed by bootstrapping with 1000 iterations [16]. Additionally, the model’s calibration was assessed using calibration curves.

## Results

A total of 286 patients were included in this study, of which 143 were case subjects and 143 were control subjects. The 286 patients were randomly divided into the training and internal validation sets in a ratio of 3:1. The training set comprised 102 case subjects and 112 control subjects who were used to develop the model and nomogram. The validation set containing 40 case subjects and 32 control subjects was used for internal validation. Table 1 lists the demographic and clinical characteristics of PTB patients in the training and validation sets. As shown, the ratio of case subjects to all subjects was comparable between the two data sets (47.7% vs 56.9%; *P* = 0.173).

**Table1:** Clinical characteristics of PTB patients in the training set and validation set

Variable	Training set(*n* = 214)	Validation set(*n* = 72)	*p*
*Pulmonary fungal infection* [*n*(%)]			
Yes	102 (47.7)	41 (56.9)	0.173
No	112 (52.3)	31 (43.1)	
*Age* [*n*(%)]			
> 65 years old	54 (25.2)	16 (22.2)	0.607
≤ 65 years old	160 (74.8)	56 (77.8)	
*Gender* [*n*(%)]			
Male	164 (76.6)	48 (66.7)	0.095
Female	50 (23.4)	24 (33.3)	
*Address* [*n*(%)]			
North	206 (96.3)	71 (98.6)	0.458
South	8 (3.7)	1 (1.4)	
*Smoke* [*n*(%)]			
Yes	89 (41.6)	29 (40.3)	0.845
No	125 (58.4)	43 (59.7)	
*Drink* [*n*(%)]			
Yes	67 (31.3)	22 (30.6)	
No	147 (68.7)	50 (69.4)	0.905
*Category* [*n*(%)]			
Initial	155 (72.4)	16 (22.2)	0.372
Recurrent	59 (27.6)	56 (77.8)	
*Drug resistance* [*n*(%)]			
No drug resistance	178 (83.2)	59 (81.9)	0.563
Single drug resistance	17 (7.9)	4 (5.6)	
Other drug resistance	19 (8.9)	9 (12.5)	
*Illness course* [*n*(%)]			
≤ 1 year	104 (48.6)	33 (45.8)	0.062
> 1 year, ≤ 5 years	59 (27.6)	13 (18.1)	
> 5 years, ≤ 10 years	19 (8.9)	14 (19.4)	
> 10 years	32 (15.0)	12 (16.7)	
*Pulmonary cavitation* [*n*(%)]			
Yes	126 (58.9)	34 (7.2)	0.085
No	88 (41.1)	38 (52.8)	
*Pleural effusion* [*n*(%)]			
Yes	82 (38.3)	25 (34.7)	0.585
No	132 (61.7)	47 (65.3)	
*Regular anti-tuberculosis* [*n*(%)]			
Yes	121 (56.5)	42 (58.3)	0.791
No	93 (43.5)	30 (41.7)	
*Broad-spectrum antibiotics were used for at least 1 week* [*n*(%)]			
Yes	89 (41.6)	35 (48.6)	0.298
No	125 (58.4)	37 (51.4)	
*Glucocorticoid were used for at least 2 weeks* [*n*(%)]			
Yes	31 (14.5)	8 (11.1)	0.470
No	183 (85.5)	64 (88.9)	
*Chemotherapy or immunosuppressants* [*n*(%)]			
Yes	10 (4.7)	1 (1.4)	0.210
No	204(95.3)	71(98.6)	
*Invasive operation* [*n*(%)]			
Yes	150 (70.1)	48 (66.7)	0.586
No	64 (29.9)	24 (33.3)	
*Surgery* [*n*(%)]			
Yes	81 (37.9)	27 (37.5)	
No	133 (62.1)	45 (62.5)	0.958
*Bacterial pneumonia* [*n*(%)]			
Yes	27 (12.6)	12 (16.7)	0.386
No	187 (87.4)	60 (83.3)	
*Chronic bronchitis/ COPD/Bronchiectasis/Asthma* [*n*(%)]			
Yes	28 (13.1)	8 (11.1)	0.662
No	186 (86.9)	64 (88.9)	
*Silicosis* [*n*(%)]			
Yes	3 (1.4)	4 (5.6)	0.070
No	211 (98.6)	68 (94.4)	
*Interstitial lung disease* [*n*(%)]			
Yes	7 (3.3)	2 (2.8)	1.000
No	207 (96.7)	70 (97.2)	
*Hepatic injury* [*n*(%)]			
Yes	68 (31.8)	18 (25.0)	
No	146 (68.2)	54 (75.0)	0.278
*Renal inadequacy* [*n*(%)]			
Yes	15 (7.0)	1 (1.4)	0.081
No	199 (93.0)	71 (98.6)	
*Tumor / Hematological disease* [*n*(%)]			
Yes	12 (5.6)	5 (6.9)	0.773
No	202 (94.4)	67 (93.1)	
*Hypoproteinemia* [*n*(%)]			
Yes	77 (36.0)	28 (38.9)	0.658
No	137 (64.0)	44 (61.1)	
*Anemia* [*n*(%)]			
Yes	62 (29.0)	21 (29.2)	0.975
No	152 (71.0)	51 (70.8)	
*Diabetes* [*n*(%)]			
Yes	49 (22.9)	19 (26.4)	0.547
No	165 (77.1)	53 (73.6)	

### Predictors entering the model

The characteristics of PTB patients with and without pulmonary fungal coinfection in the training set are summarized in Table 2. Age, category, illness course, pulmonary cavitation, pleural effusion, use of broad-spectrum antibiotics for at least 1 week, chemotherapy or immunosuppressants, surgery, bacterial pneumonia, chronic bronchitis or chronic obstructive pulmonary disease or bronchiectasis or asthma, hypoproteinemia, and anemia were significantly associated with pulmonary fungal coinfection, as evaluated by univariate analyses. Table 3 shows the results from the multivariate logistic analyses. The following seven factors were statistically significant: illness course, pulmonary cavitation, use of broad-spectrum antibiotics for at least 1 week, chemotherapy or immunosuppressants, surgery, bacterial pneumonia, and hypoproteinemia (Tables 2 and 3).

**Table 2:** Univariate analysis of PTB patients with pulmonary fungal coinfection (training dataset, *N* = 214)

Variable	Case group (*n* = 102)	Control group (*n* = 112)	*X*^2^ /Fisher	*P*
*Age* [*n*(%)]			3.893	0.048
> 65 years old	32 (31.4)	22 (19.6)		
≤ 65 years old	70 (68.6)	90 (80.4)		
*Gender* [*n*(%)]			0.492	0.483
Male	76 (74.5)	88 (78.6)		
Female	26 (25.5)	24 (21.4)		
*Address* [*n*(%)]				0.483
North	97 (95.1)	109 (97.3)		
South	5 (4.9)	3 (2.7)		
*Smoke* [*n*(%)]			2.401	0.121
Yes	48 (47.1)	41 (36.6)		
No	54 (52.9)	71 (63.4)		
*Drink* [*n*(%)]			0.016	0.898
Yes	32 (31.4)	35 (31.3)		
No	70 (68.6)	77 (68.7)		
*Category* [*n*(%)]			6.187	0.013
Initial	20 (19.6)	39 (34.8)		
Recurrent	82 (80.4)	73 (65.2)		
*Drug resistance *[*n*(%)]			3.999	0.135
No drug resistance	82 (80.4)	96 (85.7)		
Single drug resistance	12 (11.8)	5 (4.5)		
Other drug resistance	8 (7.8)	11 (9.8)		
*Illness course* [*n*(%)]			23.067	< 0.001
≤ 1 year	34 (33.3)	70 (62.5)		
> 1 year, ≤ 5 years	32 (31.4)	27 (24.1)		
> 5 years, ≤ 10 years	11 (10.8)	8 (7.1)		
> 10 years	25 (24.5)	7 (6.3)		
*Pulmonary cavitation *[*n*(%)]			15.043	< 0.001
Yes	74 (72.5)	52 (46.4)		
No	28 (27.5)	60 (53.6)		
*Pleural effusion *[*n*(%)]			4.966	0.026
Yes	47 (46.1)	35 (31.3)		
No	55 (53.9)	77 (68.7)		
*Regular anti-tuberculosis* [*n*(%)]			2.163	0.141
Yes				
No	63 (52.1)	58 (51.8)		
	39 (41.9)	54 (48.2)		
***Broad-spectrum****Advanced antibiotics were used for at* ***least****lest 1 week* [*n*(%)]			39.314	< 0.001
Yes	65 (63.7)	12 (21.4)		
No	37 (36.3)	24 (78.6)		
*Glucocorticoid were used for at* ***least****lest 2 weeks* [*n*(%)]			2.698	0.1
Yes	19 (18.6)	12 (10.7)		
No	83 (81.4)	100 (89.3)		
*Chemotherapy or immunosuppressants* [*n*(%)]				0.007
Yes	9 (8.8)	1 (0.9)		
No	93(91.2)	111(99.1)		
*Invasive operation* [*n*(%)]			0.556	0.456
Yes	69 (67.6)	81 (72.3)		
No	33 (32.4)	31 (27.7)		
*Surgery* [*n*(%)]			5.609	0.018
Yes	47 (46.1)	34 (30.4)		
No	55 (53.9)	78 (69.6)		
***Bacterial pneumonia****Bacterial infection in the lungs *[*n*(%)]			21.051	< 0.001
Yes	24 (23.5)	3 (2.7)		
No	78 (76.5)	109 (97.3)		
*Chronic bronchitis/ COPD/****Bronchiectasis***Bronchitis/*Asthma* [*n*(%)]			5.266	0.022
Yes	19 (18.6)	9 (8.0)		
No	83 (81.4)	103 (92.0)		
*Silicosis* [*n*(%)]				0.107
Yes	3 (2.9)	0 (0)		
No	99 (97.1)	112 (100)		
*Interstitial lung disease* [*n*(%)]				1
Yes	3 (3.9)	4 (3.6)		
No	99 (97.1)	108 (96.4)		
***Hepatic injury****Hypohepatia *[*n*(%)]			1.82	0.177
Yes	37 (36.3)	31 (27.7)		
No	65 (63.7)	81 (72.3)		
*Renal inadequacy *[*n*(%)]			0.208	0.648
Yes	8 (6.29)	7 (4.90)		
No	94 (93.71)	105 (95.10)		
*Tumor / Hematological disease* [*n*(%)]			3.808	0.051
Yes	9 (8.8)	3 (2.7)		
No	93 (91.2)	109 (97.3)		
*Hypoproteinemia *[*n*(%)]			30.289	< 0.001
Yes	56 (54.9)	21 (17.9)		
No	46 (45.1)	91 (81.2)		
*Anemia* [*n*(%)]			14.107	< 0.001
Yes	42 (41.2)	20 (17.9)		
No	60 (58.8)	92 (82.1)		
*Diabetes* [*n*(%)]			0.044	0.834
Yes	24 (23.5)	25 (22.3)		
No	78 (76.5)	87 (77.7)

**Table 3:** Multivariate analysis of PTB patients with pulmonary fungal coinfection(training dataset, *N* = 214)

Variable	*β*-coefficient	S.E	Wald	Sig	Exp(B)	95%CI
Illness course			13.867	0.003		
Illness course(1)	1.381	0.518	7.103	0.008	3.978	1.441–10.980
Illness course(2)	1.430	0.667	4.599	0.032	4.180	1.131–15.450
Illness course(3)	2.431	0.691	12.366	< 0.001	11.368	2.933–44.063
Pulmonary cavitation	1.241	0.405	9.377	0.002	3.460	1.563–7.656
Broad-spectrum antibiotics were used for at least 1 week	1.148	0.396	8.425	0.004	3.153	1.452–6.847
Chemotherapy or immunosuppressants	3.028	1.264	5.736	0.017	20.646	1.733–245.934
Surgery	0.769	0.382	4.041	0.044	2.157	1.019–4.565
Bacterial pneumonia	1.894	0.782	5.857	0.016	6.645	1.434–30.799
Hypoproteinemia	1.801	0.593	9.222	0.002	6.054	1.894–19.352

### Establishment and internal validation of the model and the nomogram

**Fig. 2:**
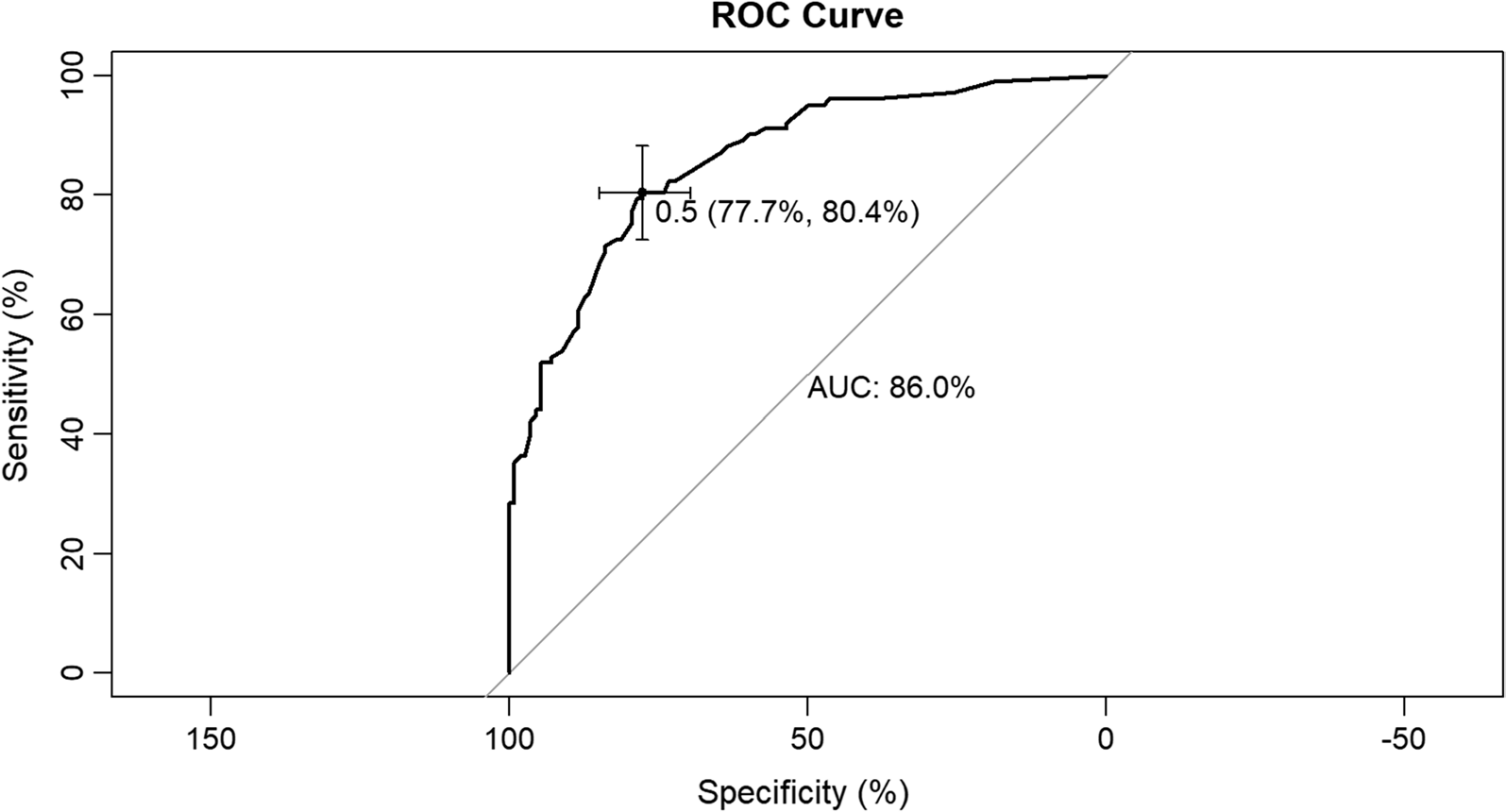
ROC curve of the established model in training set

**Fig. 3:**
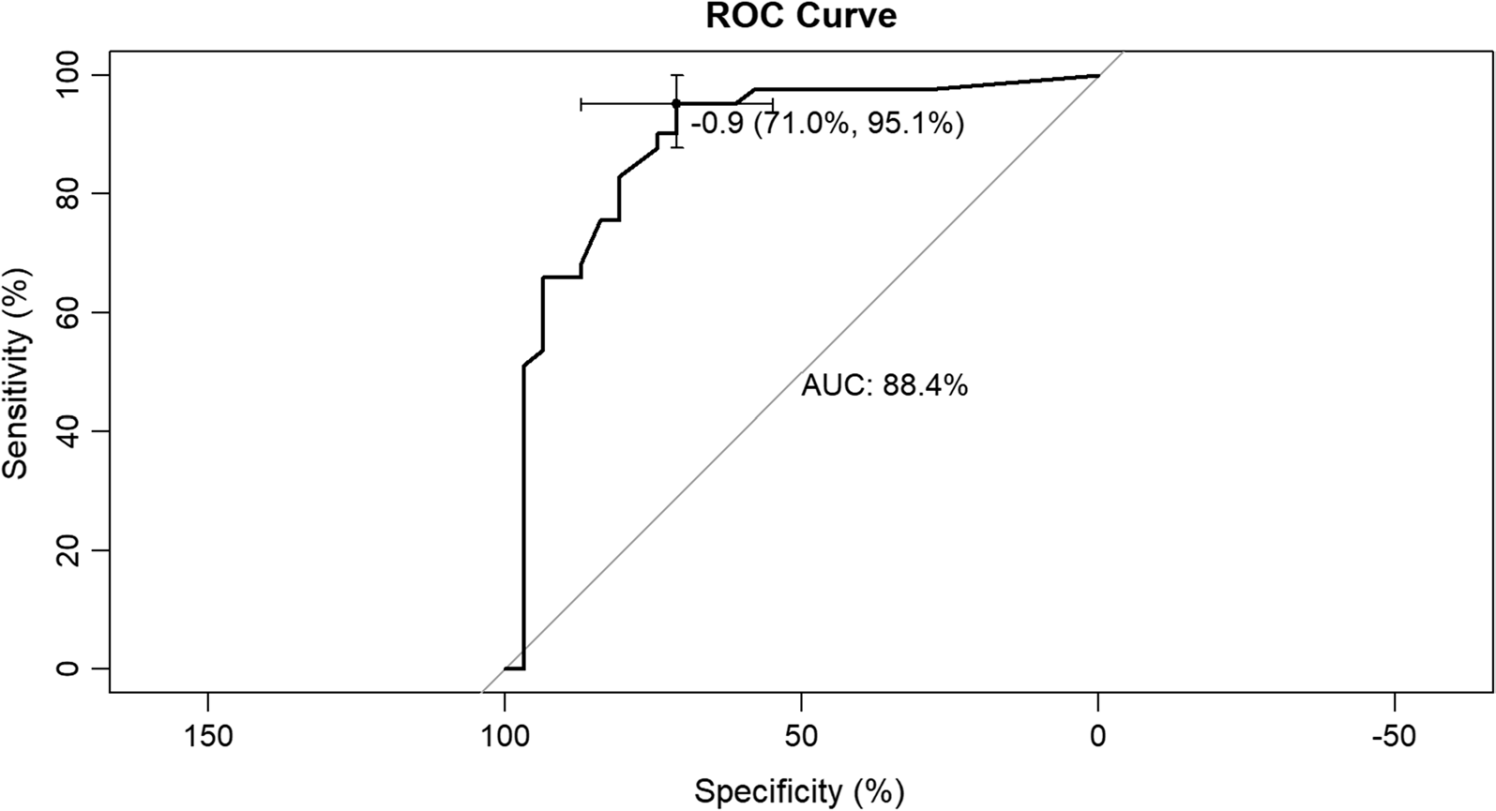
ROC curve of the established model in validation set. *ROC curve of the established model and in the internal validation. AUC (1) shows the discrimination in the model, and AUC (2) of the internal validation. ROC, receiver operating characteristic; AUC, area under the curve

**Fig. 4:**
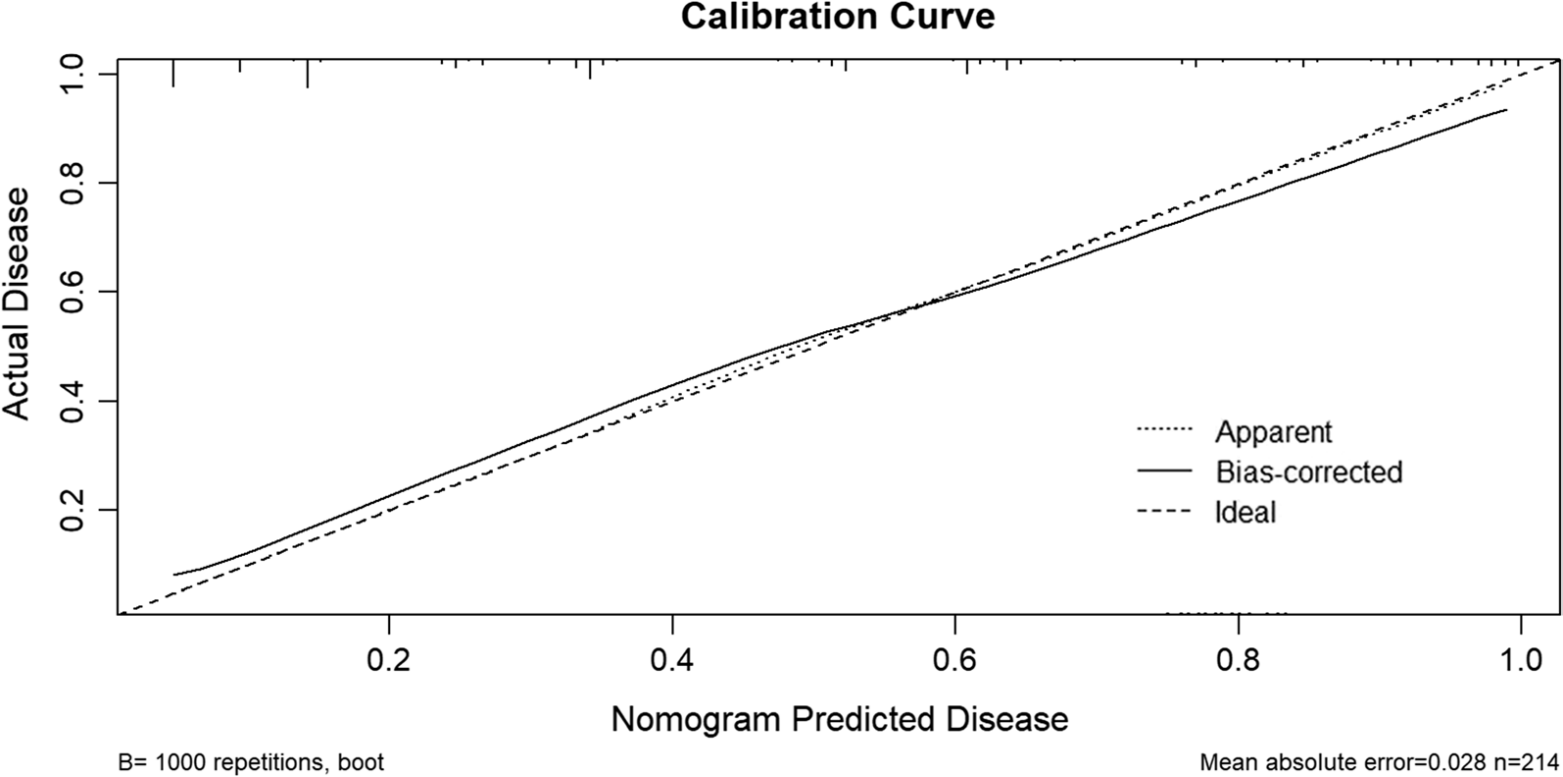
Calibration curve in training set

**Fig. 5:**
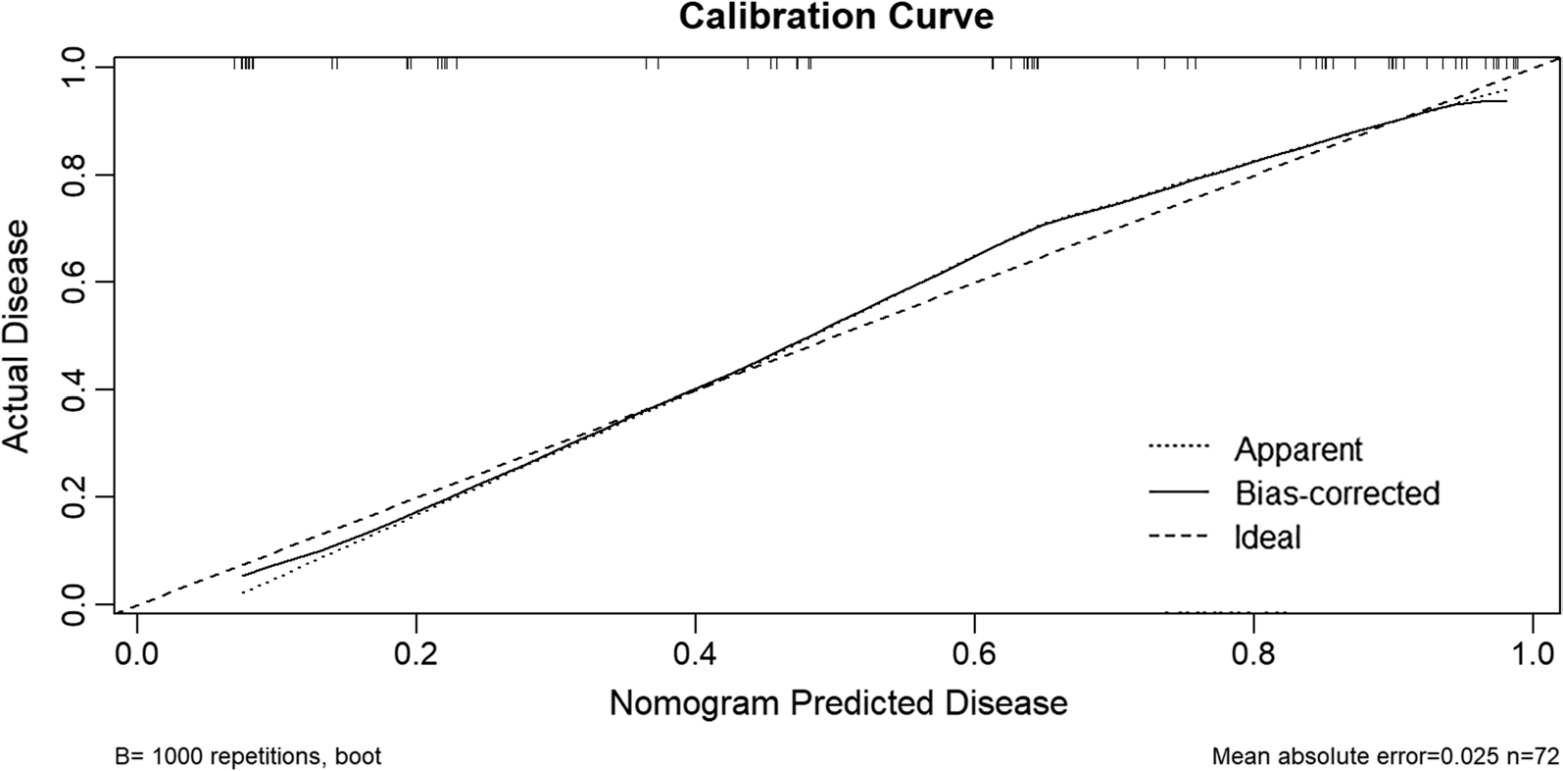
Calibration curve in validation set

We developed a predictive model for assessing the probability of pulmonary fungal coinfection in PTB patients based on the aforementioned 7 predictors. The Odds Ratios of predictors entered in the model were as follows:1 year < illness course ≤ 5 years, 3.978; 5 years < illness course ≤ 10 years, 4.180; illness course > 10 years, 11.368; pulmonary cavitation, 3.460; use of broad-spectrum antibiotics for at least 1 week, 3.153; chemotherapy or immunosuppressants, 20.646; surgery, 2.157; bacterial pneumonia, 6.645, and hypoproteinemia, 6.054. As shown in Figs. 1 and 2, the predictive model showed good discrimination in both training [area under the curve (AUC) = 0.860, 95% confidence interval (CI) = 0.811–0.909, in Fig. 2] and internal validation (AUC = 0.884, 95% CI = 0.799–0.970, in Fig. 3) sets following bootstrapping (resampling = 1000 times). The optimal cutoff was 0.5 according to the receiver operating characteristic (ROC) curve and the sensitivity and specificity were 0.777 and 0.804, respectively, in the training set (Fig. 2). Moreover, the sensitivity and specificity of the ROC curve in internal validation were 0.71 and 0.951, respectively (Fig. 3). The calibration curves also showed that the probabilities assessed using the prediction model displayed a satisfactory consistency with the actual probability for both the training and internal validation sets (Figs. 4 and 5).

**Fig. 6:**
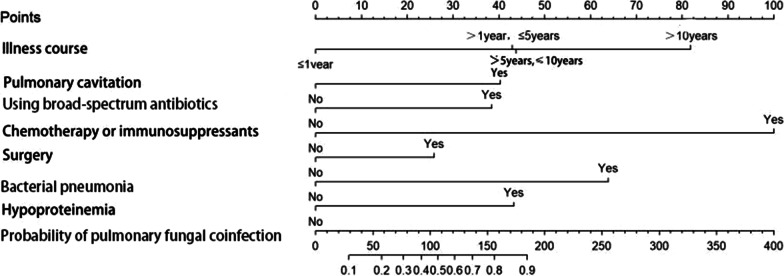
Nomogram for predicting the probability of pulmonary fungal coinfection in PTB patients. *Nomogram for predicting the probability of pulmonary fungal coinfection in PTB patients and its predictive performance. First, find the points for each predictor (variable) of a patient on the uppermost rule; then, add all points to calculate the “total points;” finally find the corresponding predicted probability of pulmonary fungal coinfection on the lowest rule.

To provide physicians with a quantitative tool for individualized prediction of pulmonary fungal coinfection, a nomogram was constructed according to the results of multivariable logistic regression (Fig. 6). The formula based on the model was as follows: Logit(P) = -2.970 + 1.381*illness course (> 1 year, ≤ 5 years) + 1.430*illness course (> 5 years, ≤ 10 years) + 2.431*illness course (> 10 years) + 1.241*pulmonary cavitation(yes = 1; no = 0) + 1.148*broad-spectrum antibiotics use for at least 1 week(yes = 1; no = 0) + 3.028*chemotherapy or immunosuppressants(yes = 1; no = 0) + 0.769*surgery(yes = 1; no = 0) + 1.894*bacterial pneumonia(yes = 1; no = 0) + 1.801*hypoproteinemia(yes = 1; no = 0).

## Discussion

People are susceptible to PTB or fungal infection when their immunity weakens. Both TB and pulmonary fungal infections increase the risk of co-infection with another disease, which may cause pulmonary disability and even death [6, 8]. For PTB patients, if pulmonary fungal coinfection is diagnosed early, it can be treated efficiently, and serious consequences can be prevented [31, 38]. Hence, coinfection with PTB and pulmonary fungal infection should be considered. An accurate predictive model is imperative for clinicians and PTB patients. Although several clinical characteristics have been proposed previously, models predicting the probability of pulmonary fungal coinfection for PTB patients are lacking. No corresponding model predicting the probability of combined pulmonary fungal infection in PTB patients has been reported to date. Considering these reasons, in this real-world retrospective study, a multivariable model based on easily accessible clinical parameters was developed and internally validated.

Clinical characteristics of PTB patients with pulmonary fungal coinfection were associated with the illness course, pulmonary cavitation, use of broad-spectrum antibiotics for at least 1 week, chemotherapy or immunosuppressants, surgery, bacterial pneumonia, and hypoproteinemia, consistent with the findings of some previous studies [10, 15–17, 19–25, 27, 28]. Patients with a long illness course may suffer from recurrence and more severe lung tissue destruction, resulting in pulmonary fungal coinfection. In line with our study, Page et al. found that pulmonary cavitation was a predictor of fungal pulmonary coinfection [29, 30]. These cavities form an ideal culture plate for the fungi by providing plenty of oxygen along with necrotic tissue material, stimulating the occurrence of pulmonary fungal coinfection. The use of broad-spectrum antibiotics and bacterial pneumonia not only breaks the balance of airway microecology but also reduces airway resistance to fungi. Hypoproteinemia and chemotherapy or immunosuppressant use were also significant variables predictive of pulmonary fungal coinfection in PTB patients, likely because these factors may decrease immunity, thus making the patients more susceptible to fungal coinfection. Although our data also showed that age, recurrent TB, and invasive operations were significant predictors in the univariate analysis [10, 15–17, 27], these were subsequently excluded as they were insignificant according to multivariate logistic regression analysis. The use of glucocorticoids has been identified as a potential variable in some studies [10, 15–17, 25–27], while our findings differ, probably because of the limited number of cases (*n* = 31), and warrant further investigation. Drug resistance was also observed as a possible predictor in previous research [5, 10, 15–17, 27] but this was statistically insignificant herein.

A nomogram was developed using the above predictors for facilitating individualized prediction of pulmonary fungal coinfection. For example, using the nomogram, we found that a PTB patient with pulmonary cavitation and an illness course of more than 1 year but no more than 5 years, had a 30% likelihood of pulmonary fungal coinfection. The nomogram can help clinicians improve decision-making for these patients.

However, there are some limitations to our study. First, the training set was randomly selected from all PTB patients leading to lower evidence strength and failure in showing the incidence rate. Second, this was a single-center retrospective study, which inevitably suffered from confounding biases. Third, our predictive model is diagnostic but diagnosis requires some auxiliary examinations and is correspondingly time-consuming. The time for collecting outcome variables (maybe several days after hospital admission) sometimes may be several days compared to the predictors (hospital admission), contributing to a lower accuracy of the prediction model. Finally, only internal validation was performed to evaluate the discrimination ability and calibration of the scoring model, and external validation is warranted to confirm the performance of the nomogram (Additional file 1).

## Conclusion

In conclusion, a prediction model, comprising seven independent predictors, may empower clinicians and PTB patients for timely and early assessment and inform them of the likelihood of pulmonary fungal coinfection more precisely, facilitating better management. Its favorable calibration and discrimination might permit it to be suitable for routine clinical practice in most hospitals.

## Supplementary Information

**Additional file 1.** The diagnostic criteria for pulmonary fungal infection.

## Data Availability

The datasets generated and/or analyzed during the current study are not publicly available due to a privacy-protection policy, but are available from the corresponding author on reasonable request.
