# Supplementary material for: Clinical characteristics and predictive model of pulmonary tuberculosis patients with pulmonary fungal coinfection
Source: BMC Pulm Med. 2023 Feb 7;23:56. doi: 10.1186/s12890-023-02344-4 (PMC9903523; doi:10.1186/s12890-023-02344-4)
Supplement: Supplementary file 1 — Additional file 1. The diagnostic criteria for pulmonary fungal infection. [file 12890_2023_2344_MOESM1_ESM.pdf]

Here is the instruction of the diagnostic criteria for pulmonary fungal infection in the "Expert Consensus on the Diagnosis and Treatment of Pulmonary Mycosis" proposed by the Infections Group of the Respiratory Society of the Chinese Medical Association in 2007：

1. At least have one host incidence risk factor:(1) Peripheral blood leukocytes <0.5 * 10 ^ 9 / L,neutropenia or deficiency, duration > 10d; (2) Body temperature> 38℃ or <36℃, accompanied by one of the following conditions: ① persistent neutropenia within 60d (10d); ② Previously received or was currently receiving immunosuppressant therapy within 30d; ③ A history of invasive fungal infection; ④AIDS patient; ⑤ GVHD; ⑥ Continuous application of corticosteroids for more than 3 weeks; ⑦ Chronic underlying diseases; ⑧ Trauma, major surgery, long-term ICU stay, long mechanical ventilation, indwelling catheter, total parenteral nutrition and long-term use of broad-spectrum antibiotics (either one).

2. Meet the clinical characteristics of pulmonary mycosis:(1) Main clinical features: 1. Invasive pulmonary aspergillosis: chest X-ray and CT examination found increased subpleural nodules or halo symptoms around the lesions in the early stages of the disease; chest X-ray and CT examination found pulmonary cavity or a crescent sign 10-15days after onset; 2. Pneumocyte pneumonia: chest CT examination showed hair glass lung interstitial infiltration. (2) Secondary clinical features: 1. Continuous fever> 96h, no significant improvement after aggressive antibiotic treatment; 2. Symptoms and signs of lung infection: cough, expectoration, hemoptysis, chest pain, dyspnea, moist rales or pleural friction sound; 3. Imageological examination shows new non-specific lung infiltration other than the main clinical features.

3. Have lung histopathological evidence or any microbiological evidence：(1) Direct microscopy of the endotracheal attractor or qualified sputum samples found the hyphae, and the same fungus was isolated for two consecutive times in the culture.；(2) BALF was detected by direct microscopy with positive fungal culture.；(3) Qualified sputum or BALF direct microscopy or culture found that Cryptococcus neoformans.；(4) Crycoccus capsular polysaccharide antigen was positive by latex coagulation.；(5) The G-test was positive for two consecutive times.；(6) The GM-test was positive for two consecutive times.
